# Context matters: examining factors influencing the implementation of evidence-based family systems care for small and sick newborns across the care continuum

**DOI:** 10.3389/frhs.2025.1383292

**Published:** 2025-04-10

**Authors:** Christina Schuler, Faith Agbozo, Emmanuel Bansah, Barbara Preusse-Bleuler, Richard Owusu, Riccardo E. Pfister

**Affiliations:** ^1^ Institute of Global Health, Faculty of Medicine, University of Geneva, Geneva, Switzerland; ^2^ Department of Health Sciences, Institute of Nursing, Zurich University of Applied Sciences, Winterthur, Switzerland; ^3^ Department of Family and Community Health, Fred. N. Binka School of Public Health, University of Health and Allied Science, Ho, Ghana; ^4^ Department of Health Information, Volta Regional Hospital, Hohoe, Ghana; ^5^ Neonatal and Pediatric Intensive Care Unit, University Hospitals of Geneva and Geneva University, Geneva, Switzerland

**Keywords:** context assessment, continuum of care, family-centered care, Ghana, maternal and neonatal care, implementation science, small and sick newborns

## Abstract

**Introduction:**

The health and wellbeing of small and sick newborns and their families can be enhanced through family systems care (FSC) along the care continuum. FSC encompasses a broader approach than family-centered care. FSC identifies individual and family strengths while acknowledging illness-related suffering and providing expertise to help soften it through relational family systemic interventions. Contextual factors of the targeted healthcare setting need to be understood to implement FSC. This study aims to assess healthcare professionals’ perceptions of health system features that may influence the successful context-adapted implementation of FSC into the care continuum for small and sick newborns and their families in the Ghanaian healthcare setting.

**Methods:**

Cross-sectional data were collected from 143 healthcare professionals, comprising nurses, midwives, and physicians who provide maternal and newborn care at a secondary facility and 13 primary health facilities in the Hohoe Municipality, Ghana. The Context Assessment for Community Health (COACH) instrument, employing Likert scales ranging from 1 to 5 and including questions on training history, was used to collect data on FSC through self-administered interviews. Data were analyzed using descriptive statistics with STATA.

**Results:**

While 48.9% of healthcare professionals reported never receiving any didactic or school-based training, the majority (96.5%) indicated a need for in-service training in FSC. From the highest score of 5, the COACH dimension for *organizational resources* had the lowest score (2.8). *Community engagement*, *commitment to work*, *monitoring services for action*, and *informal payment* reported scores between 3.7 and 3.9. The highest scores were reported for the *leadership* and *work culture* dimensions, at 4.1 and 4.2, respectively. Among the different units of the care continuum, the largest variations were observed in the subdimensions of *organizational resources* (2.5–3.4) and *informal payment* (3.6–4.4).

**Conclusion:**

The COACH tool provided contextual guidance for developing training strategies to implement a contextually appropriate FSC program in Ghana, which is likely to be adaptable and relevant in other low- and middle-income countries. Healthcare professionals perceive themselves as committed, with a favorable work culture and a positive perception toward their leaders, but they report limited resources and challenges in accessing knowledge sources. These findings indicate a readiness for FSC training along the continuum of care in the perinatal period.

## 1 Introduction

Every year, nearly 30 million small or sick newborns are at risk of dying or experiencing disability; almost all (98%) neonatal deaths occur in low- and middle-income countries (LMICs) (1). “Small newborns” weigh <2,500 g at birth, including preterm and low-birth-weight newborns, while “sick newborns” have medical or surgical conditions (2).

Family members of small and sick newborns are at high risk of facing long-term psychosocial hardships, which can impede the neurodevelopmental, social, and cognitive growth of their offspring (3–5). Small and sick newborns require resources that economically burden families and governments in LMICs, straining healthcare systems and national budgets in both the short and long term (5–7). The high disease burden has caused the World Health Organization (WHO) to identify care for small and sick newborns as a global priority (2, 8).

Ensuring access to high-quality, nurturing healthcare across a structured continuum of care (CoC) that links hospitals, primary care, and communities with home care is a necessity to improve newborn health and family wellbeing. Nurturing care for newborns must start during pregnancy. The WHO recommends at least eight informative and supportive antenatal contacts (9). Nurturing care should be continued during intrapartum and postnatal care in healthcare facilities or hospitals and later at home (3, 10). However, in Ghana and many LMICs, families are often inadequately involved in healthcare services along the CoC (11–13).

Family-centered care (FCC) is a widely recognized approach in maternal, newborn, and child health that involves the family in patient care by implementing policies such as open visiting hours, family participation in bedside care, and improved communication. While family-centered care acknowledges the importance of the family, its implementation in the healthcare system is often inconsistent, and its adoption remains unclear in many settings (14, 15).

Family systems care (FSC) builds upon the principles of FCC but adopts a broader, systemic approach. FSC actively involves families in caring for their small and sick newborns from the onset of pregnancy through birth and postnatal care. Differing from FCC, which primarily focuses on policies within healthcare settings, FSC also integrates a societal structure into the CoC, emphasizing the interaction and reciprocity between multiple systems, including the family, society, and the healthcare system (16). Grounded in six theoretical foundations—postmodernism, systems theory, cybernetics, communication and change theory, and the biology of cognition—FSC provides a structured framework to guide healthcare professionals (17–20). It employs the Calgary Family Models, both the assessment model (CFAM) and the intervention model (CFIM), to identify individual and family strengths while addressing illness-related suffering through systemic interventions (17, 21).

By focusing on cognitive, emotional, and behavioral domains, FSC supports families and healthcare professionals in a targeted, sustainable care approach that extends beyond the hospital setting (14, 17). It aims at providing sustained neurodevelopmental and nurturing care, particularly for small and sick newborns (10, 17, 22). Furthermore, FSC delivers an avenue for newborns and their families to receive respectful care and acknowledges their human rights, addressing a global concern in this respect highlighted by Isaacs (23) and Rosa-Mangeret et al. (24).

However, the FSC concept has not yet been tailored to resource-limited settings and collectivistic societies, such as Ghana (15, 22, 25, 26), nor has it been adopted along the CoC (27). To improve the health and wellbeing of small and sick newborns and their families, FSC needs to include health professionals in an evidence-based and locally adapted context (8). It is, therefore, unavoidable to understand the contextual factors of the targeted healthcare setting. Only then can an FSC program be planned and implemented; it needs to anticipate potential challenges when integrating into a team, department, or organization (28, 29).

Our study had two objectives: first, to determine the training history in FSC of healthcare professionals that provide maternal and newborn healthcare along the CoC at both hospital and primary (community) care levels, and second, within the same Hohoe Municipality of Ghana, to evaluate perceptions of the healthcare professionals of local health system building blocks that may influence the implementation of an FSC program in the CoC for pregnant women, small and sick newborns, and their families.

## 2 Materials and methods

### 2.1 Study design

An online cross-sectional survey was performed between June 2023 and August 2023.

### 2.2 Study settings

The study was conducted in the Hohoe Municipality, situated in the Volta Region of Ghana. The majority of the population engages in crop and livestock farming and petty trading (30, 31). The municipality has a secondary-level referral hospital (regional level II hospital) where advanced care is possible. Primary care services in the municipality are delivered through two approaches: (1) eight health centers and clinics and (2) five community-based health planning and services (CHPS) zones, each equipped with a dedicated health facility structure known as a CHPS compound.

The regional level II hospital serves over 200,000 inhabitants. It provides antenatal care, basic and emergency obstetric services, and essential newborn care. It includes a post-delivery ward and neonatal intensive care unit (NICU) for small and sick newborns. The wards are staffed by general nurses, a couple of specialist pediatric nurses, midwives, pediatricians, obstetricians/gynecologists, house officers, and other general physicians. The newly established telecommunication unit at the Volta Regional Hospital coordinates referrals between the various care units. The hospital also has a recovery ward for postsurgical care of women who have undergone cesarean sections. The public health and nutrition unit (PHNU) located in the hospital offers child welfare clinic services, such as growth monitoring, vaccinations, and nutrition counseling. In addition, it sometimes acts as a liaison between the NICU and primary care facilities (32). CHPS and health centers provide basic maternal, child, and neonatal healthcare, along with adult healthcare services. Neonatal care includes breastfeeding support, growth monitoring, vaccinations, management of minor ailments, and referrals to higher-level facilities (33). The CHPS and health centers are run by community health nurses and enrolled nurses who typically receive a shorter education compared to general nurses. The team is occasionally complemented by midwives, physician assistants, and community volunteers.

### 2.3 Participants and eligibility criteria

Healthcare professionals working with pregnant or laboring women and/or small and sick newborns and their families were included in the study. Nurses, midwives, physician assistants, and physicians of all levels were eligible. The study participants worked either at the antenatal care clinic, labor ward, recovery ward, postnatal ward, neonatal intensive care unit, and the public health and nutrition unit of hospitals or at one of the 13 primary care facilities. Students undergoing basic vocational training in healthcare were excluded from the survey. Although community health volunteers play a role in the maternal and newborn care continuum in Ghana, we chose not to include them in this study because our planned FSC training was designed specifically for healthcare professionals with formal training. In Ghana, community health volunteers do not receive training in FCC or FSC and would, therefore, be unable to answer all survey questions. In addition, financial and logistical constraints prevented us from including lay personnel in this survey.

### 2.4 Study size

The sample size was estimated with a population census approach following Israeli (34). Since the population was small, we decided to use the entire population. At the time of the study, 208 healthcare professionals were working across various units, from the hospital to the primary care level.

### 2.5 Data collection instruments

In brief, data collection was based on four validated scales. Here, we report on the Context Assessment for Community Health (COACH) instrument (35) and additional questions related to training history (36). The other three scales, which investigated implementation outcomes and healthcare professionals’ attitudes and skills toward family systems care, will be reported separately.

To quantitatively assess contextual factors, Bergström et al. (35) developed the COACH instrument, a validated, theory-based tool to evaluate healthcare contexts in LMICs. The COACH instrument is based on the interconnected building blocks of the WHO and the context dimension of the “Integrated Promoting Action on Research Implementation in Health Services” (I-PARIHS) framework (35, 37).

The I-PARIHS framework focuses on contextual factors (28, 38), including local characteristics such as leadership style, culture, past experience with change, mechanisms for embedding change, and routine methods of providing feedback on performance (38, 39).

The COACH instrument is suitable for nurses, midwives, and physicians at both hospital and community levels and serves three functions: (1) enhancing opportunities to act on locally identified shortcomings of the health system to increase effectiveness; (2) structuring the planning, adaptation, and promotion of implementation strategies in the local context; and (3) linking contextual characteristics to outcome indicators of healthcare interventions (35).

### 2.6 Training history in FSC

Questions on training history were adapted from Baumann et al. (36). In our survey, we treated the terms “family-centered” (FCC) and “family systems care” (FSC) as equivalent, although FSC extends beyond the scope of FCC (14). We made this choice to minimize potential confusion, as study participants were somewhat familiar with the concept of FCC but unlikely able to differentiate it from FSC.

### 2.7 COACH questionnaire on healthcare systems context

The validated COACH instrument consists of 49 questions across eight dimensions (Table 1). The original English version of the COACH instrument was used with permission from the author (35). The questions were minimally adapted to fit our context. For instance, the original “Members of the unit approach clients with respect” was transformed to “Members of the unit approach pregnant women/small or sick babies and their families with respect.”

**Table 1:** Definitions of COACH dimension.

Dimension subdimension	Definition^a^
Organizational resources *Human resources, space, communication and transport, medicine and equipment, financing*	The availability of recourses that allow an organization (unit) to adapt successfully to internal and external pressures
Community engagement	The mutual communication, deliberation, and activities that occur between community members and an organization (unit)
Monitoring services for action	The process of using locally derived data to assess performance and plan how to improve outcomes in an organization (unit)
Commitment to work	The individual's identification with and involvement in a particular organization (unit)
Work culture *Culture of learning and change, culture of responsibility*	The way “we do things” in an organization (unit) reflects a supportive work culture
Leadership	The actions of a formal leader in an organization (unit) to influence change and excellence in practice achieved through clarity and engagement
Informal payment *Informal payment, nepotism, accountability*	Payments or benefits given to individual(s) in an organization (unit), which are made outside the officially accepted arrangement, to acquire an advantage or service
Sources of knowledge	The availability and use of sources of knowledge in an organization (unit) to facilitate best practice

^a^ Unit refers to the ward in the hospital or the primary care level facility where the respondent works.

Participants were asked to rate their level of agreement using a five-point Likert scale for all items, except for items in the *sources of knowledge* dimension, where the 0–1 scale was used. The scoring of dimensions ranged from 1, denoting “strongly disagree,” to 5, denoting “strongly agree.” For the subdimension *informal payment*, the first six items were reverse-scored. For the dimension *sources of knowledge*, scores were assigned as follows: 0 denoting “not available, never/rarely,” 0.5 denoting “occasionally,” and 1 denoting “frequently/always.”

The data collection instrument was pretested in a different municipality within the Volta Region of Ghana, involving hospital and community-experienced healthcare professionals. Based on their responses, we added an explanatory introduction to our context analysis.

### 2.8 Data processing and statistical analysis

Data were collected using Kobo Collect (Kobo Collect, 2023), an open-source program for collecting survey data. The questionnaire was self-administered. Hospital and district management provided us with the contacts of the in-charges (supervisors) of the different care units. An online survey link was shared with these in-charges at both the hospital and primary care levels, who then disseminated it to eligible participants of the various work units through existing WhatsApp groups.

Data were downloaded from Kobo Collect into a single master file (Microsoft Excel 2016) and de-identified, with socio-demographic variables aggregated to prevent re-identification. Stata Version 17 was used for analysis. The data were individually checked for consistency and appropriateness. Descriptive statistics such as means, standard deviations, frequencies, and proportions were used to analyze data. We report demographics using frequencies and proportions. The overall results for dimensions and subdimensions were largely dichotomized into strong agreement and agreement vs. disagreement, strong disagreement, or neutral.

The COACH dimensions for each unit of care and combined across the care continuum are reported as means with corresponding standard deviation (SD). Cronbach's *α*, a measure of reliability and internal consistency, was calculated for the eight dimensions.

### 2.9 Ethical considerations

Ethical clearance was obtained from the Ghana Health Service Ethical Review Committee (GHS-ERC 027/03/23). The Volta Regional Hospital Management and the Hohoe Municipal Health Directorate granted permission to conduct the study. The study adhered to the Declaration of Helsinki (40). Study information was provided via the online survey link, and participants gave informed consent online prior to participation. The survey was conducted confidentially. While some demographic information was collected, no personally identifiable data were recorded or linked to individual responses. All data were stored securely.

## 3 Results

### 3.1 Socio-demographic characteristics

A total of 208 healthcare professionals were deemed eligible at the time of data collection. One hundred forty-three healthcare professionals participated in the study (68.8% response rate). Among the respondents were pediatric nurses (2.1%), general nurses (21%), midwives (35.7%), medical assistants (0.7%), physician assistants (0.7%), and general physicians (1.4%). The majority of healthcare professionals were women (80.4%), about 67.1% were aged between 30 and 39 years, and most had either a diploma (48.6%) or a certificate (32.4%). Few had a bachelor's degree (14.8%), and only 2.1% had a master's degree. In addition, 70.6% had less than 4 years of work experience in their specific unit of care (Table 2).

**Table 2:** Health responders’ demographics along the continuum of care [*N* (%)].

Variable	Group	Overall	ANC	Labor ward	Recovery ward	NICU	Postnatal ward	PHNU	Health centers	CHPS
		*N* = 143	*N* = 10	*N* = 17	*N* = 2	*N* = 17	*N* = 9	*N* = 8	*N* = 54	*N* = 26
Age (years)	20–29	36 (25.2)	2 (20.0)	1 (5.9)	0 (0.0)	4 (23.5)	3 (33.3)	1 (12.5)	20 (37.0)	5 (19.2)
	30–39	96 (67.1)	7 (70.0)	15 (88.2)	2 (100)	13 (76.5)	5 (55.7)	6 (75.0)	29 (53.7)	19 (73.1)
	≥40	11 (7.7)	1 (10.0)	1 (5.9)	0 (0.0)	0 (0.0)	1 (11.1)	1 (12.5)	5 (9.3)	2 (7.7)
Gender	Female	115 (80.4)	10 (100)	16 (94.1)	2 (100)	11 (64.7)	8 (88.9)	7 (87.5)	42 (77.8)	19 (73.1)
Profession	CHN	38 (26.6)	0 (0.0)	0 (0.0)	0 (0.0)	0 (0.0)	0 (0.0)	4 (50.0)	17 (31.5)	17 (65.4)
	General nurse	30 (21.0)	0 (0.0)	0 (0.0)	2 (100.0)	11 (64.7)	1 (11.1)	0 (0.0)	14 (25.9)	2 (7.7)
	Enrolled nurse	16 (11.2)	3 (30.0)	0 (0.0)	0 (0.0)	2 (11.8)	0 (0.0)	3 (37.5)	5 (9.3)	3 (11.5)
	Pediatric nurse	3 (2.1)	0 (0.0)	0 (0.0)	0 (0.0)	3 (17.7)	0 (0.0)	0 (0.0)	0 (0.0)	0 (0.0)
	Midwife	51 (35.7)	7 (70.0)	16 (94.1)	0 (0.0)	0 (0.0)	8 (88.9)	1 (12.5)	16 (29.6)	3 (11.5)
	Medical staff^a^	5 (3.5)	0 (0.0)	1 (5.9)	0 (0.0)	1 (5.9)	0 (0.0)	0 (0.0)	2 (3.7)	1 (3.8)
Education	Certificate	47 (32.4)	0 (0.0)	0 (0.0)	0 (0.0)	3 (17.7)	1 (11.1)	3 (37.5)	20 (37.7)	19 (73.1)
	Diploma	69 (48.6)	6 (60.0)	9 (52.9)	1 (50.0)	10 (58.8)	6 (66.7)	3 (37.5)	28 (52.8)	6 (23.1)
	Bachelor	21 (14.8)	1 (10.0)	7 (41.2)	1 (50.0)	2 (11.8)	2 (22.2)	2 (25.0)	5 (9.4)	1 (3.8)
	Master’s	3 (2.1)	2 (20.0)	0 (0.0)	0 (0.0)	1 (5.9)	0 (0.0)	0 (0.0)	0 (0.0)	0 (0.0)
	Other	3 (2.1)	1 (10.0)	1 (5.9)	0 (0.0)	1 (5.9)	0 (0.0)	0 (0.0)	0 (0.0)	0 (0.0)
Work experience (years)	0–3	101 (70.6)	10 (100.0)	8 (47.1)	2 (100.0)	13 (76.5)	6 (66.7)	3 (37.5)	40 (74.1)	19 (73.1)
	4–9	37 (25.9)	0 (0.0)	8 (47.1)	0 (0.0)	4 (23.5)	3 (33.3)	5 (62.5)	11 (20.4)	6 (23.1)
	10+	5 (3.5)	0 (0.0)	1 (5.9)	0 (0.0)	0 (0.0)	0 (0.0)	0 (0.0)	3 (5.5)	1 (3.8)

ANC, antenatal care; CHN, community health nurse; NICU, neonatal intensive care unit; PHNU, public health and nutrition unit; CHPS, community-based health planning and services.

^a^ Medical staff included two general physicians, two physician assistants, and one medical assistant.

### 3.2 Family systems care training—history

About half (48.9%) of the healthcare professionals had participated in didactic or school-based training on family systems care: 30.8% in the previous year and 20.3% prior to that. The majority (76.9%) had never participated in long-term (>1-month course) training on family systems care, and 74.8% had never received on-the-job training or supervision. Only 12.6% had long-term training in the previous year, while 10.5% had done so earlier. The need for training in family systems care was affirmed by 96.5% of participants (Table 3).

**Table 3:** Training history in family systems (centered) care along the continuum of care [*N* (%)].

Variable	Overall	ANC	Labor ward	Recovery ward	NICU	Postnatal ward	PHNU	Health centers	CHPS
	*N* = 143	*N* = 10	*N* = 17	*N* = 2	*N* = 17	*N* = 9	*N* = 8	*N* = 54	*N* = 26
Have you received didactic or school-based training on family systems (centered) care?									
No, never	70 (48.9)	4 (40.0)	5 (29.4)	0 (0.0)	8 (47.1)	3 (33.3)	4 (50.0)	28 (51.8)	18 (69.2)
Yes, before the past year	29 (20.3)	2 (20.0)	5 (29.4)	0 (0.0)	7 (41.2)	0 (0.0)	0 (0.0)	10 (18.5)	5 (19.2)
Yes, in the past year	44 (30.8)	4 (40.0)	7 (41.2)	2 (100)	2 (11.7)	6 (66.7)	4 (50.0)	16 (29.6)	3 (11.5)
Have you received a structured long-term (>1-month course) training on family systems (centered) care?									
No, never	110 (76.9)	7 (70.9)	12 (70.6)	2 (100.0)	10 (58.8)	8 (88.9)	5 (62.5)	46 (85.2)	20 (76.9)
Yes, before the past year	15 (10.5)	2 (20.0)	3 (17.6)	0 (0.0)	4 (23.5)	0 (0.0)	1 (12.5)	3 (5.5)	2 (7.7)
Yes, in the past year	18 (12.6)	1 (10.0)	2 (11.8)	0 (0.0)	3 9 (17.8)	1 (11.1)	2 (25.0)	5 (9.3)	4 (15.4)
Have you received on-the-job training, in-service training, or supervision on family system (centered) care?									
No, never	107 (74.8)	5 (50.0)	12 (70.6)	2 (100.0)	15 (88.2)	6 (66.7)	4 (50.0)	44 (81.5)	19 (73.1)
Yes, before the past year	11 (7.7)	2 (20.0)	2 (11.8)	0 (0.0)	1 (5.9)	0 (0.0)	1 (12.5)	2 (3.7)	3 (11.5)
Yes, in the past year	25 (17.5)	3 (30.0)	3 (17.6)	0 (0.0)	1 (5.9)	3 (33.3)	3 (37.5)	8 (14.8)	4 (15.4)
Do you feel there is a need for additional training on family system (centered) care									
Yes	138 (96.5)	10 (100.0)	17 (100.0)	2 (100.0)	17 (100.0)	8 (88.9)	8 (100.0)	52 (96.3)	24 (92.3)

ANC, antenatal care; NICU, neonatal intensive care unit; PHNU, public health and nutrition unit; CHPS, community-based health planning and services.

### 3.3 Overall description of the eight dimensions and subdimensions

#### 3.3.1 Organizational resources

Human resources were deemed sufficient in numbers and adequacy of training and skills to complete all requested tasks by 55.2% of healthcare professionals. Two-thirds (60.8%) agreed that there were enough staff with the right training and skills to do their job in the best possible way, 40.6% agreed to have sufficient space to provide healthcare services in their units, and 61.5% reported adequate access to communication tools (e.g., telephones or radios). Also, 44.8% of healthcare professionals reported disposing of enough medicine; 44.7% agreed that their unit had enough functional medical equipment, such as a thermometer and pulse oximeter, about half (55.2%) agreed to have enough disposables, such as syringes, needles, and gloves; and 44.7% reported that they received additional resources such as medicine when the workload increased. However, 69.9% disagreed with having easy access to transport and/or fuel needed to provide healthcare. Regarding financing, 66.5% disagreed that their unit received funding according to an established financial plan.

#### 3.3.2 Community engagement

The majority of healthcare professionals reported asking community members about the quality of healthcare services (72.7%), listening to their replies to this question (83.2%), and holding community meetings to discuss health matters (59.4%). In addition, 72.0% reported encouraging community members and 72.4% encouraging other organizations to improve healthcare.

#### 3.3.3 Monitoring services for action

A majority of healthcare professionals (83.9%) agreed that they received regular updates on their unit's performance based on data, and 81.8% reported that this information/data was regularly discussed during formal, scheduled meetings. Also, 86.0% agreed to use locally collected data to make plans to improve health outcomes in their units. Regular monitoring activities were reported by 85.3% to be done by comparing the unit’s work with its action plan, and 85.3% of respondents acknowledged that their units benchmarked with national or international guidelines.

#### 3.3.4 Commitment to work

Regarding commitment to work, 80.4% agreed that they were proud to work in their unit, 66.4% reported being satisfied to work in their unit, and 81.8% felt encouraged to perform at their best.

#### 3.3.5 Work culture

Regarding work culture, 94.4% of healthcare workers were willing to adopt new healthcare practices based on guidelines and recommendations, 81.1% agreed their unit helped them in improving and developing personal skills, and 91.6% felt encouraged to seek new information on healthcare practices. Also, 95.8% agreed their unit worked for the wellbeing of clients/patients, and 92.3% felt personally responsible for improving healthcare services.

#### 3.3.6 Leadership

Among respondents, 85.3% reported trusting their unit leader and 83.2% reported that their leader handles stressful situations calmly. Also, 85.3% answered that their leader actively listens, acknowledges, and then responds to requests and concerns; a majority also noted that their leader effectively resolves conflicts (83.9%), encourages the introduction of new ideas and practices (88.8%), and takes initiatives to make things happen in the unit (75.5%).

#### 3.3.7 Informal payment

Most healthcare professionals disagreed (94.6%) that clients/patients were always required to make informal payments for access to healthcare, and 93.7% denied that treatment started earlier when such informal payments were made. Most professionals (96.5%) opposed the sale of medicines or equipment to clients/patients when these items were meant to be free. Healthcare professionals also disagreed that healthcare services were prioritized for friends and family (95.1%) and similarly disagreed that jobs or other benefits were provided to friends and family first (93.7%). Healthcare professionals reported that efforts were made to stop informal payments for appropriate healthcare services (60.2%) and to ask for informal payments (62.9%).

#### 3.3.8 Sources of knowledge

Most healthcare professionals (83.2%) acknowledged the availability of clinical practice guidelines in their field of practice (maternal and newborn care, including the care of high-risk pregnancies and deliveries and care of small and sick newborns). Out of the 87.4% who had received in-service training, workshops, or courses, 35.2% participated never or rarely (0–5 times a month), 40.0% participated occasionally (6–10 times a month), and 24.8% participated frequently or always (11 or more per month). However, only 37.1% of the 143 respondents had access to the Internet at their workplace (Table 4).

**Table 4:** Descriptive values of dimensions and subdimensions of the COACH questionnaire (*N* = 143).

Dimension and subdimension	Mean	Median	Strongly disagree *n* (%)	Disagree *n* (%)	Neutral *n* (%)	Agree *n* (%)	Strongly agree *n* (%)
(1) Organizational resources							
Human resources							
This facility has enough workers with the right training and skills to do everything that needs to be done	3.21	4.00	12 (8.4)	41 (28.7)	11 (7.7)	62 (43.4)	17 (11.8)
This facility has enough workers with the right training and skills to do their job in the best possible way	3.38	4.00	11 (7.7)	29 (20.3)	16 (11.2)	68 (47.5)	19 (13.3)
Space							
My unit has enough space to provide healthcare services	2.78	2.00	28 (19.6)	45 (31.4)	12 (8.4)	46 (32.2)	12 (8.4)
Communication and transport							
My unit has access to the transport and fuel that are needed to provide healthcare	2.18	2.00	49 (34.2)	51 (35.7)	13 (9.1)	27 (18.9)	3 (2.1)
My unit has access to the communication tools (e.g., telephones or radios) that are needed to provide maternal and newborn healthcare services	3.34	4.00	16 (11.2)	27 (18.9)	12 (8.4)	68 (47.5)	20 (14.0)
Financing							
My unit receives money according to an established financial plan	2.25	2.00	44 (30.8)	51 (35.7)	20 (14.0)	24 (16.8)	4 (2.7)
My unit has money that we can decide how to use	1.83	2.00	66 (46.1)	51 (35.7)	13 (9.1)	10 (7.0)	3 (2.1)
Medicines and equipment							
My unit has enough medicine to provide healthcare services	3.06	3.00	10 (6.9)	43 (30.1)	26 (18.2)	55 (38.5)	9 (6.3)
My unit has enough functional equipment, such as a thermometer and pulse oximeter	2.97	3.00	18 (12.6)	48 (33.6)	13 (9.1)	47 (32.8)	17 (11.9)
My unit has enough disposable medical equipment, such as syringes, needles, gloves	3.22	4.00	13 (9.1)	35 (24.5)	16 (11.2)	65 (45.4)	14 (9.8)
If the workload increases, my unit can get additional resources, such as medicine	3.00	1.20	18 (12.6)	38 (26.6)	23 (16.1)	53 (37.1)	11 (7.6)
(2) Community engagement							
In my unit, we ask community members what they think about the healthcare service	3.63	4.00	8 (5.6)	18 (12.6)	13 (9.1)	83 (58.0)	21 (14.7)
In my unit, we listen to what community members think about the healthcare service	3.91	4.00	2 (1.4)	9 (6.3)	13 (9.1)	94 (65.7)	25 (17.5)
In my unit, we have meetings with community members to discuss health matters	3.46	4.00	12 (8.4)	22 (15.4)	24 (16.8)	59 (41.2)	26 (18.2)
In my unit, we encourage community members to contribute to improving the health	3.76	4.00	6 (4.2)	15 (10.5)	19 (13.3)	70 (48.9)	33 (23.1)
In my unit, we encourage other organizations to contribute to improving the health	3.67	4.00	6 (4.2)	16 (11.3)	20 (14.1)	76 (53.5)	24 (16.9)

Dimension Subdimension Question	Mean	Median	Strongly disagree *n* (%)	Disagree *n* (%)	Neutral *n* (%)	Agree *n* (%)	Strongly agree *n* (%)
(3) Monitoring services for action							
I receive regular updates about my unit's performance based on information/data	3.97	4.00	4 (2.8)	6 (4.2)	13 (9.1)	86 (60.1)	34 (23.8)
My unit discusses information/data from our unit in a regular, formal way, such as in regularly scheduled meetings	3.89	4.00	5 (3.5)	9 (6.3)	12 (8.4)	87 (60.8)	30 (21.0)
My unit regularly uses unit information/data to make plans for improving its health	3.99	4.00	6 (4.2)	5 (3.5)	9 (6.3)	87 (60.8)	36 (25.2)
My unit regularly monitors its work by comparing it with the unit's action plans	4.04	4.00	4 (2.8)	4 (2.8)	13 (9.1)	82 (57.3)	40 (28.0)
My unit regularly compares its work with national or other guidelines	4.00	4.00	5 (3.5)	4 (2.8)	12 (8.4)	86 (60.1)	36 (25.2)
(4) Commitment to work							
I am proud to work in this unit	3.96	4.00	4 (2.8)	10 (7.0)	14 (9.8)	74 (51.7)	41 (28.7)
I am satisfied to work in this unit	3.56	4.00	9 (6.3)	20 (14.0)	19 (13.3)	71 (49.6)	24 (16.8)
I feel encouraged to do my very best at work.	4.05	4.00	5 (3.5)	6 (4.2)	15 (10.5)	67 (46.8)	50 (35.0)
(5) Work culture							
Culture of learning and change							
My unit is willing to use new healthcare practices, such as guidelines and recommendations	3.96	4.00	2 (1.4)	3 (2.1)	3 (2.1)	79 (55.2)	56 (39.2)
My unit helps me to improve and develop my skills	3.97	4.00	5 (3.5)	7 (4.9)	15 (10.5)	76 (53.1)	40 (28.0)
I am encouraged to seek new information on healthcare practices.	4.11	4.00	4 (2.8)	2 (1.4)	6 (4.2)	92 (64.3)	39 (27.3)
Culture of responsibility							
My unit works for the good of the clients	4.32	4.00	4 (2.8)	0 (0)	2 (1.4)	77 (53.8)	60 (42.0)
Members of the unit feel personally responsible for improving healthcare service	4.21	4.00	4 (2.8)	2 (1.4)	5 (3.5)	81 (56.6)	51 (35.7)
Members of the unit approach clients with respect	4.32	4.00	4 (2.8)	0 (0)	2 (1.4)	77 (53.8)	60 (42.0)
(6) Leadership							
I trust the unit leader	4.12	4.00	1 (0.7)	5 (3.5)	15 (10.5)	76 (53.1)	46 (32.2)
The leader handles stressful situations calmly	4.09	4.00	1 (0.7)	6 (4.2)	17 (11.9)	74 (51.7)	45 (31.5)
The leader actively listens, acknowledges, and then responds to requests and concerns	4.09	4.00	4 (2.8)	4 (2.8)	13 (9.1)	75 (52.4)	47 (32.9)
The leader effectively resolves any conflicts that arise	4.04	4.00	3 (2.1)	7 (4.9)	13 (9.1)	78 (54.5)	42 (29.4)
The leader encourages the introduction of new ideas and practices	4.13	4.00	3 (2.1)	4 (2.8)	9 (6.3)	81 (56.6)	46 (32.2)
The leader makes things happen	3.85	4.00	6 (4.2)	8 (5.6)	21 (14.7)	74 (51.7)	34 (23.8)
(7) Informal payment							
Informal payment							
Clients must always give informal payments to health workers to access healthcare	4.62	5.0	104 (72.7)	32 (22.4)	2 (1.4)	3 (2.1)	2 (1.4)
Clients are treated more quickly when they make informal payments to health worker	4.61	5.0	101 (70.6)	33 (23.1)	5 (3.5)	3 (2.1)	1 (0.7)
Medicines or equipment that should be available for free to clients have been sold in my unit	4.71	5.0	110 (76.9)	28 (19.6)	3 (2.1)	1 (0.7)	1 (0.7)
Health workers are sometimes absent from work, earning money at other places	4.64	5.0	105 (73.4)	31 (21.7)	2 (1.4)	4 (2.8)	1 (0.7)
Nepotism							
Health workers in my unit give healthcare services to friends and family first	4.56	5.0	93 (65.0)	43 (30.1)	2 (1.4)	4 (2.8)	1 (0.7)
Health workers in my unit give jobs or other benefits to friends and family first	4.58	5.0	98 (68.5)	36 (25.2)	4 (2.8)	4 (2.8)	1 (0.7)
Accountability							
Efforts are made to stop clients from providing informal payment to get appropriate healthcare services	3.28	4.0	30 (20.9)	14 (9.8)	13 (9.1)	57 (39.9)	29 (20.3)
Efforts are made to stop health workers from asking clients for informal payment	3.34	4.0	28 (19.6)	15 (10.5)	10 (7.0)	59 (41.2)	31 (21.7)

Sources of knowledge						
Non-scaled dimension	Mean	Median	Yes *n* (%)	Never/rarely *n* (%)	Occasionally *n* (%)	Frequently/always *n* (%)
(8) Sources of knowledge						
Clinical practice guidelines for maternal/newborn care	0.79	1.0	119 (83.2)	8 (6.7)	32 (26.9)	79 (66.4)
Other printed material for work (e.g., textbooks, journals)	0.69	1.0	103 (72.0)	15 (14.6)	33 (32.0	55 (53.4)
In-service training/workshops/courses available	0.38	0.5	125 (87.4)	44 (35.2)	50 (40.0)	31 (24.8)
Internet available to you at your unit	0.81	1.0	53 (37.1)	5 (9.4)	10 (18.9)	38 (71.7)
Electronic decision support (e.g., mobile, phone, application)	0.83	1.0	97 (67.8)	9 (9.3)	14 (14.4)	74 (76.3)

### 3.4 Description of the COACH subdimensions across units along the care continuum

Table 5 presents the mean and standard deviations for the subdimension across units.

(1) The organizational resources dimension had a mean score of 2.8, indicating that healthcare professionals disagreed on sufficient resources. In the recovery ward, PHNU, and health centers, the scores were 3.4, 3.0, and 3.0, respectively, suggesting a neutral view.
(2) For community engagement, the mean score was 3.7, indicating that healthcare professionals were more favorable than neutral about the commitment of their units toward community involvement. The highest scores were observed at the primary care level, with a score of 4.1 in the health centers and 3.9 in the CHPS compounds.
(3) Monitoring services for action received an overall mean score of 3.9, suggesting that healthcare professionals agree that health institutions use locally driven data to evaluate health units’ performance and provide action plans for maternal and newborn care. The lowest scores, each at 3.6, were reported in the ANC and labor ward, while the highest scores, at 4.2, were observed in the NICU and PHNU.
(4) For commitment to work, the mean score was 3.7, indicating that healthcare professionals were committed to their work. The recovery ward had the highest score of 4.5, whereas the other units scored between 3.5 and 4.1.
(5) Work culture had a mean score of 4.2, suggesting a positive attitude toward learning and change and a high feeling of responsibility. There were only small variations across the CoC, with the highest scores in the recovery ward (4.6) and health centers (4.4) and the lowest scores in the ANC and CHPS (3.9).
(6) The leadership dimension had a mean score of 4.1, indicating agreement with their current leadership across the care continuum. The lowest score was reported at the CHPS level (3.6).
(7) Informal payments had a mean score of 3.9, suggesting that healthcare professionals were critical to elements of informal payments comprising nepotism and accountability. Across the care continuum, the PHNU and the ANC scored the lowest, at 3.6 and 3.8, respectively.
(8) For source of knowledge, the mean score on a scale from 0 to 1 was 0.7.

**Table 5:** Overall mean ± SD and by unit for the COACH subdimensions across units along the care continuum.

Scales	Overall	ANC	Labor ward	Recovery ward	NICU	Postnatal ward	PHNU	Health centers	CHPS
	*N* = 143	*N* = 10	*N* = 17	*N* = 2	*N* = 17	*N* = 9	*N* = 8	*N* = 54	*N* = 26
(1) Organizational resources	2.8 ± 0.7	2.8 ± 0.6	2.5 ± 0.5	3.4 ± 0.3	2.8 ± 0.7	2.7 ± 0.3	3.0 ± 0.8	3.0 ± 0.7	2.7 ± 0.5
Human resources	3.3 ± 1.1	3.0 ± 1.1	2.1 ± 0.9	4. 0 ± 0.0	3.1 ± 1.2	2.7 ± 1.0	3.7 ± 1.0	3.8 ± 0.9	3.3 ± 1.2
Space	2.8 ± 1.3	2.8 ± 1.4	3.1 ± 1.4	2.5 ± 0.7	3.1 ± 1.3	1.9 ± 0.9	2.0 ± 1.1	3.1 ± 1.2	2.4 ± 1.5
Communication and transport	2.8 ± 0.9	2.7 ± 0.9	3.1 ± 0.8	3.6 ± 0.4	2.9 ± 0.9	3.2 ± 0.9	2.8 ± 1.2	2.6 ± 1.0	2.3 ± 0.8
Financing	2.4 ± 1.0	2.2 ± 0.9	1.4 ± 0.6	2.0 ± 0.9	1.6 ± 0.8	1.4 ± 0.5	2.4 ± 1.2	2.4 ± 1.0	2.0 ± 0.8
Medicines and equipment	3.1 ± 0.9	3.0 ± 0.6	2.9 ± 0.9	3.7 ± 0.4	3.0 ± 0.6	3.2 ± 0.7	3.5 ± 0.9	3.1 ± 0.9	2.9 ± 0.7
(2) Community engagement	3.7 ± 0.8	3.2 ± 0.6	3.2 ± 0.7	2.9 ± 0.1	3.2 ± 0.8	3.2 ± 0.6	3.6 ± 0.7	4.1 ± 0.6	3.9 ± 0.8
(3) Monitoring services for actions	3.9 ± 0.8	3.6 ± 0.9	3.6 ± 0.8	4.1 ± 0.9	4.2 ± 0.5	4.0 ± 0.9	4.2 ± 0.7	4.1 ± 0.6	3.8 ± 0.9
(4) Commitment to work	3.7 ± 0.9	3.7 ± 0.7	3.5 ± 1.1	4.5 ± 0.2	3.9 ± 0.8	3.5 ± 1.1	4.1 ± 0.7	4.1 ± 0.8	3.7 ± 0.8
(5) Work culture	4.2 ± 0.7	3.9 ± 1.1	4.0 ± 0.8	4.6 ± 0.6	4.3 ± 0.5	4.3 ± 0.4	4.3 ± 0.7	4.4 ± 0.5	3.9 ± 0.8
Culture of learning and change	4.1 ± 0.7	3.9 ± 1.0	3.9 ± 0.8	4.5 ± 0.7	4.3 ± 0.5	4.2 ± 0.5	4.3 ± 0.7	4.2 ± 0.6	3.8 ± 0.8
Culture of responsibility	4.3 ± 0.7	3.9 ± 1.1	4.1 ± 0.9	4.7 ± 0.5	4.4 ± 0.4	4.5 ± 0.4	4.3 ± 0.7	0.5 ± 0.5	3.9 ± 0.9
(6) Leadership	4.1 ± 0.7	4.0 ± 0.7	3.9 ± 0.8	4.5 ± 0.7	4.3 ± 0.4	4.3 ± 0.5	4.2 ± 0.6	4.2 ± 0.8	3.6 ± 0.8
(7) Informal payment	3.9 ± 0.5	3.8 ± 0.7	3.9 ± 0.7	4.4 ± 0.2	4.2 ± 0.4	3.9 ± 0.3	3.6 ± 0.4	4.0 ± 0.5	3.9 ± 0.6
Accountability	3.3 ± 1.4	3.0 ± 1.5	3.2 ± 1.4	4.5 ± 0.7	3.6 ± 1.4	2.9 ± 1.3	3.0 ± 1.1	3.6 ± 1.4	2.9 ± 1.4
Informal payment	4.5 ± 0.8	4.5 ± 0.9	4.5 ± 0.9	5.0 ± 0.7	4.9 ± 0.2	4.4 ± 0.7	4.0 ± 0.5	4.5 ± 0.8	4.3 ± 0.9
Nepotism	4.6 ± 0.7	4.5 ± 0.9	4.5 ± 0.7	5.0 ± 0.7	4.9 ± 0.2	4.6 ± 0.6	4.1 ± 0.6	4.5 ± 0.7	4.5 ± 0.9
(8) Sources of knowledge (scale 0 to 1)	0.7 ± 0.3	0.6 ± 0.3	0.7 ± 0.2	0.7 ± 0.3	0.8 ± 0.2	0.6 ± 0.3	0.6 ± 0.1	0.8 ± 0.2	0.5 ± 0.3

ANC, antenatal care; NICU, neonatal intensive care unit; PHNU, public health and nutrition unit; CHPS, community-based health planning and services.

Likert scales 0–5, except for item (8).

### 3.5 Internal reliability of the COACH questionnaire

Overall, the COACH instrument showed a very good to high internal consistency (Cronbach's *α* range: 0.713–0.793), with all dimensions exceeding the accepted standard for satisfactory internal reliability of >0.70. The source of knowledge dimension had the highest Cronbach's α estimate (0.793), while the monitoring services for action dimension had the lowest (0.713) estimate (Table 6).

**Table 6:** Cronbach's *α* estimates for the different COACH dimensions.

Dimension	Items	Score range	Cronbach's *α*
Organizational resources	11	1–5	0.780
Community engagement	5	1–5	0.762
Monitoring services for action	5	1–5	0.713
Commitment to work	3	1–5	0.721
Work culture	6	1–5	0.719
Leadership	6	1–5	0.744
Informal payment	8	1–5	0.787
Sources of knowledge	5	0–1	0.793

## 4 Discussion

This study had two objectives: to map the contextual factors influencing care for families of small and sick newborns along the care continuum in the Hohoe Municipality of Ghana and to determine the training history of healthcare professionals in family-centered or FSC.

For training history, the main finding was that half of the healthcare professionals along the care continuum for small and sick newborns had received school-based training in FSC and that an overwhelming majority expressed the need for increased and/or continuous training. A general lack of multidisciplinary training opportunities in maternal and neonatal care in LMICs has been reported by Bolan et al. (41). The lack of training opportunities in FSC has also been demonstrated in Ghana and similar settings despite its high potential to improve maternal and pediatric care (42–44).

The COACH instrument allowed a detailed mapping of the FSC components along the continuum of care. Five of the eight dimensions had a high mean score, indicating context features supportive of change. However, organizational resources (dimension 1) scored low and showed the highest variability between the different units of care, pointing out insufficient resources to adequately perform required tasks. Unreliable or non-functional resources for maternal and newborn care have been reported by several other studies in East Africa (45, 46), Southern Africa (47), and sub-Saharan Africa in a systematic review (48). Unsurprisingly, the lack of resources in CHPS has been reported frequently (32, 49, 50). Unreliable resources present an undeniable barrier to the implementation of evidence-based interventions (51). Yet, in a cross-sectional multinational study involving 4,300 health facilities, some healthcare professionals continued to provide substandard care despite the availability of resources such as amenities, equipment, medications, and evidence-based guidelines (46). Transportation and fuel appear to be critical elements, although not all units across the continuum of care may have the same reasons. Hospital-based units rely on ambulance services for referrals, a known bottleneck in the Hohoe municipality (32) and other parts of Ghana (52). At the primary care level, the lack of transportation limits healthcare professionals from conducting home visits (50).

The scores for community engagement (dimension 2) were slightly higher at the primary care level (health centers and CHPS) than at the hospital level, possibly due to closer community involvement at this care level. The importance of strong community engagement has been highlighted by Elsey et al. (50), who recommended continuous support, as community involvement is declining and at risk of reverting to a more traditional health post-operation mode (53).

The high scores for monitoring services for action (dimension 3) across the care continuum suggest general satisfaction of healthcare professionals with up-to-date performance data and comparison with national or other guidelines. This contrasts with recent reports from Ghana and Malawi, where healthcare professionals were often unaware of national and international action plans, and maternal and newborn health guidelines were poorly translated into practice (32, 54). However, insufficient awareness of guidelines may paradoxically contribute to higher scores, as healthcare professionals may believe in knowing guidelines without having in-depth knowledge—or even the existence—of relevant guidelines. Adequate dissemination of guidelines and tracking their translation into practice, therefore, remain important educational targets.

Healthcare professionals reported that locally collected data were available in Ghana (55) and that the quality of maternal and newborn care data was sufficient for use in daily health decision-making.

Commitment to work (dimension 4) varied along the care continuum, with the lowest scores observed in the ANC, labor, and postnatal wards and CHPS compounds. Commitment to work was reportedly undermined by low salaries, poorly equipped facilities, insufficient human resources, and emotionally challenging situations, particularly within maternal healthcare (56, 57). We and others have recently provided evidence from Ghana suggesting that commitment to work in maternal and newborn care can be positively influenced by supportive supervision and leadership (32, 57).

Poor teamwork and work culture have been generally associated with difficulties in implementing change (35, 58). In this regard, our high overall score for work culture (dimension 5) is promising for implementing new interventions into the care continuum. Given the variations in work culture across different care units, we will have to pay particular attention to some specific wards, such as ANC and CHPS, when implementing our future FSC program.

The leadership dimension (dimension 6) has scored high across the care continuum. The lowest score in the CHPS compounds may have resulted from the specific leadership structure at the community level. In any case, evidence suggests that high leadership engagement and mentoring are favorable for the implementation of new evidence-based maternal and newborn care (59) and family-focused care (60).

Compared to studies from Rwanda (36) and Mozambique (47), the informal payment dimension (dimension 7), which represents corruption, scored slightly lower in our survey and showed greater variation between different units. Although social desirability might still have played a role in our study in that responders chose to provide answers they believe are acceptable, the online format of our survey may have warranted better anonymity, facilitating honest answers. In Ghana, two other recent studies also reported informal payments in the healthcare system (61, 62). Dalaba et al. (62) even reported that informal payments were the main non-medical costs for families during childbirth.

The low scores for knowledge sources (dimension 8) and the request for FSC training confirm our earlier findings that healthcare professionals and managers acknowledge a lack of in-service training and courses, especially at the primary care level (32). A multi-country analysis found that, beyond limited access to evidence-based guidelines and information, factors such as lack of awareness of this evidence, outdated training curricula, and unavailable hands-on training also hinder the use of evidence-based information (63).

To our knowledge, this is the first study to apply the COACH questionnaire in a West African country and the first to use the instrument to assess maternal and newborn health across the care continuum. We did not face any unexpected challenges and observed a low Cronbach's *α* in the knowledge dimension, as reported previously (36, 47). We, therefore, can confirm good overall validity of the COACH instrument and its applicability in LMICs.

### 4.1 Limitations

The understanding of the concept of family systems care and family-centered care might vary between participants and thus influence their responses. In addition, the annual reshuffling of staff, which is more common in the hospital context, most likely influences responses from these sectors. Finally, the low numbers of respondents in some units may have made the answers more subject to individual opinions. However, we believe that the validated COACH instrument proved effective and reflects current real-life conditions by providing a snapshot of the local health system features through the perspectives of their healthcare professionals. Due to the study design and constraints, particularly the long duration of the survey, some healthcare professionals may have refused to participate. However, we did not collect the reasons for refusal.

### 4.2 Implications of findings

Our findings pinpoint essential elements for long-term capacity building to strengthen the FSC approach in the Hohoe Municipality of Ghana, with its final goal to improve the health of small and sick newborns and their families along the continuum of care—from antenatal to community-based services. Using the COACH instrument, we identified the following priorities: (1) to further detail context dimensions with low scores, high variability, and potential bias, for instance, through focus groups and interviews; (2) to improve family systems care features to fit the specific context by targeting identified weaknesses in collaboration with health professionals and family members; and (3) to monitor implementation of family systems care along the care continuum with a standardized tool. The next step will be to develop a formalized qualitative interview guide based on our findings. The aim is to standardize interviews with healthcare professionals across the care continuum for families with small and sick newborns to further strengthen quantitative findings.

The COACH instrument proved suitable for assessing the healthcare context prior to implementing new interventions, and it was adapted with easy and demonstrated high internal reliability in our LMIC context. As the COACH instrument is used for the first time in West Africa for maternal and newborn care across the CoC, confirmation studies should be performed. We believe that the COACH instrument is transferable to other LMIC countries. A French translation could confirm our findings and support implementation efforts in Francophone LMICs.

The thorough context assessment aligns with the recent national health policy of Ghana (64), which aims to strengthen family and social support, and may thus be used by policymakers to scale up at the national level.

## 5 Conclusion

The COACH instrument provided contextual guidance for the development of training strategies to implement a context-adapted family systems care program in Ghana that is most likely to be adaptable and relevant in other LMICs. The contextual dimensions explored in relation to family systems care identified domains of strengths and weaknesses of the local Ghanaian health system. Healthcare professionals reported a high overall commitment to work and had a favorable work culture and supportive leadership while acknowledging challenges with resources and access to knowledge sources. These findings indicate a readiness for FSC training along the continuum of care in the perinatal period.

## Data Availability

The raw data supporting the conclusions of this article will be made available by the authors without undue reservation.

## References

[1] WHO. Survive and Thrive: Transforming Care for Every Small and Sick Newborn. Geneva: World Health Organization (2019).

[2] WHO. Standards for Improving Quality of Care for Small and Sick Newborns in Health Facilities. Geneva: World Health Organization (2020).

[3] WHO, UNICEF. Thematic Brief: Nurturing Care for Every Newborn. Geneva: World Health Organization (2021). Available at: https://nurturing-care.org/nurturing-care-for-every-newborn/

[4] Moxon SG Lawn JE Dickson KE Simen-Kapeu A Gupta G Deorari A Inpatient care of small and sick newborns: a multi-country analysis of health system bottlenecks and potential solutions. BMC Pregnancy Childbirth. (2015) 15(Suppl 2):S7. 10.1186/1471-2393-15-S2-S7 26391335 PMC4577807

[5] Ashorn P Ashorn U Muthiani Y Aboubaker S Askari S Bahl R Small vulnerable newborns—big potential for impact. Lancet. (2023) 401(10389):1692–706. 10.1016/S0140-6736(23)00354-9 37167991

[6] Lawn JE Ohuma EO Bradley E Idueta LS Hazel E Okwaraji YB Small babies, big risks: global estimates of prevalence and mortality for vulnerable newborns to accelerate change and improve counting. Lancet. (2023) 401(10389):1707–19. 10.1016/S0140-6736(23)00522-6 37167989

[7] Enweronu-Laryea CC Andoh HD Frimpong-Barfi A Asenso-Boadi FM. Parental costs for in-patient neonatal services for perinatal asphyxia and low birth weight in Ghana. PLoS One. (2018) 13(10):e0204410. -e. 10.1371/journal.pone.0204410 30312312 PMC6185862

[8] WHO. WHO Recommendations on Maternal and Newborn Care for a Positive Postnatal Experience. Geneva: World Health Organization (2022. Licence: CC BY-NC-SA 3.0 IGO. 35467813

[9] WHO. WHO Recommendations on Antenatal Care for a Positive Pregnancy Experience. Geneva: World Health Organization (2016). Available at: https://www.who.int/publications/i/item/9789241549912 28079998

[10] Black MM Behrman JR Daelmans B Prado EL Richter L Tomlinson M The principles of nurturing care promote human capital and mitigate adversities from preconception through adolescence. BMJ Glob Health. (2021) 6(4):e004436. 10.1136/bmjgh-2020-004436 33875519 PMC8057542

[11] Horwood C Haskins L Luthuli S McKerrow N. Communication between mothers and health workers is important for quality of newborn care: a qualitative study in neonatal units in district hospitals in South Africa. BMC Pediatr. (2019) 19(1):496. 10.1186/s12887-019-1874-z 31842824 PMC6913017

[12] Sakuma S Yasuoka J Phongluxa K Jimba M. Determinants of continuum of care for maternal, newborn, and child health services in rural Khammouane, Lao PDR. PLoS One. (2019) 14(4):e0215635. 10.1371/journal.pone.0215635 31013303 PMC6478320

[13] Schuler C Waldboth V Ntow GE Agbozo F. Experiences of families and health professionals along the care continuum for low-birth weight neonates: a constructivist grounded theory study. J Adv Nurs. (2023) 79(5):1840–55. 10.1111/jan.15566 36762678

[14] Bell JM. Family nursing is more than family centered care. J Fam Nurs. (2013) 19(4):411–7. 10.1177/1074840713512750 24227014

[15] Abukari AS Schmollgruber S. Concepts of family-centered care at the neonatal and paediatric intensive care unit: a scoping review. J Pediatr Nurs. (2023) 71:e1–e10. 10.1016/j.pedn.2023.04.005 37120388

[16] Bell JM. Family systems nursing: re-examined. J Fam Nurs. (2009) 15(2):123–9. 10.1177/1074840709335533 19423766

[17] Shajani Z Snell D. Wright and Leahey’s Nurses and Families A Guide to Family Assessment & Intervention. 8th ed. Philadelphia: F.A. DAVIS (2023).

[18] Whitchurch GG Constantine LL. Systems Theory. Sourcebook of Family Theories and Methods: A Contextual Approach. New York, NY, US: Plenum Press (1993). p. 325–55.

[19] Bertalanffy Lv Sutherland JW. General systems theory: foundations, developments, applications. IEEE Trans Syst Man Cybern. (1974) SMC-4(6):592–2. 10.1109/TSMC.1974.4309376

[20] Maturana HR Varela FJ. The Tree of Knowledge: The Biological Roots of Human Understanding. Boston, MA: New Science Library/Shambhala Publications (1992).

[21] Wright LM Leahey M. Nurses and Families: A Guide to Family Assessment and Intervention. 6th ed. Philadelphia: F.A Davis Company (2013).

[22] Ndwiga C Warren CE Okondo C Abuya T Sripad P. Experience of care of hospitalized newborns and young children and their parents: a scoping review. PLoS One. (2022) 17(8):e0272912. 10.1371/journal.pone.0272912 36037213 PMC9423633

[23] Isaacs D. What is the value of a human baby? J Paediatr Child Health. (2002) 38(6):608–9. 10.1046/j.1440-1754.2002.00071.x 12410877

[24] Rosa-Mangeret F Benski AC Golaz A Zala PZ Kyokan M Wagner N 2.5 million annual deaths-are neonates in low- and middle-income countries too small to be seen? A bottom-up overview on neonatal morbi-mortality. Trop Med Infect Dis. (2022) 7(5):64. 10.3390/tropicalmed7050064 35622691 PMC9148074

[25] Aubel J Martin SL Cunningham K. Introduction: a family systems approach to promote maternal, child and adolescent nutrition. Matern Child Nutr. (2021) 17(S1):e13228. 10.1111/mcn.13228 34241950 PMC8269145

[26] Ohene LA Power KJ Raghu R. Health professionals’ perceptions and practice of family centred care for children injured in road traffic accidents: a qualitative study in Ghana. J Pediatr Nurs. (2020) 53:e49–e56. 10.1016/j.pedn.2020.02.005 32113734

[27] Kokorelias KM Gignac MAM Naglie G Cameron JI. Towards a universal model of family centered care: a scoping review. BMC Health Serv Res. (2019) 19(1):564. 10.1186/s12913-019-4394-5 31409347 PMC6693264

[28] Nilsen P Bernhardsson S. Context matters in implementation science: a scoping review of determinant frameworks that describe contextual determinants for implementation outcomes. BMC Health Serv Res. (2019) 19(1):189. 10.1186/s12913-019-4015-3 30909897 PMC6432749

[29] Powell BJ Beidas RS Lewis CC Aarons GA McMillen JC Proctor EK Methods to improve the selection and tailoring of implementation strategies. J Behav Health Serv Res. (2017) 44(2):177–94. 10.1007/s11414-015-9475-6 26289563 PMC4761530

[30] Ghana Statistical Service. Composite Budget for 2020-2023 Programme Based Budget Estimates for 2020 - Hohoe Municipal Assembly. Accra: Ministry of Finance (2019). Available at: https://mofep.gov.gh/sites/default/files/composite-budget/2020/VR/Hohoe.pdf

[31] Ministry of Food and Agriculture. Hohoe Municipal. Accra: Minstry of Food and Agriculture (2023). Available at: https://mofa.gov.gh/site/directorates/district-directorates/volta-region/281-hohoe-municipal

[32] Schuler C Agbozo F Ntow GE Waldboth V. Health-system drivers influencing the continuum of care linkages for low-birth-weight infants at the different care levels in Ghana. BMC Pediatr. (2023) 23(1):501. 10.1186/s12887-023-04330-5 37798632 PMC10552361

[33] Ministry of Health. National Community-Based Health Planning and Services (CHPS) Policy. Accra: Ministry of Health Ghana (2016).

[34] Israeli G. Determining Sample Size. Gainesville, FL: University of Florida (1992). p. 1–5. Available at: https://www.psycholosphere.com/Determining%20sample%20size%20by%20Glen%20Israel.pdf

[35] Bergström A Skeen S Duc DM Blandon EZ Estabrooks C Gustavsson P Health system context and implementation of evidence-based practices—development and validation of the context assessment for community health (COACH) tool for low-and middle-income settings. Implement Sci. (2015) 10(1):120. 10.1186/s13012-015-0305-2 26276443 PMC4537553

[36] Baumann AA Hooley C Goss CW Mutabazi V Brown AL Schechtman KB Exploring contextual factors influencing the implementation of evidence-based care for hypertension in Rwanda: a cross-sectional study using the COACH questionnaire. BMJ Open. (2021) 11(9):e048425. 10.1136/bmjopen-2020-048425 34548353 PMC8458329

[37] De Savigny D Adam T. Systems Thinking for Health Systems Strengthening. Geneva: Alliance for Health Policy and Systems Research & World Health Organization (2009).

[38] Harvey G Kitson A. PARIHS Re-Visited: Introducing the I-PARIHS Framework. Implementing Evidence-Based Practice in Healthcare. London: Routledge (2015). p. 25–46.

[39] Kitson AL Harvey G. Methods to succeed in effective knowledge translation in clinical practice. J Nurs Scholarsh. (2016) 48(3):294–302. 10.1111/jnu.12206 27074390

[40] World Medical Association. World Medical Association Declaration of Helsinki: Ethical Principles for Medical Research Involving Human Subjects. Ferney-Voltaire: World Medical Association (2024). Available at: https://www.wma.net/what-we-do/medical-ethics/declaration-of-helsinki/

[41] Bolan N Cowgill KD Walker K Kak L Shaver T Moxon S Human resources for health-related challenges to ensuring quality newborn care in low- and middle-income countries: a scoping review. Glob Health Sci Pract. (2021) 9(1):160–76. 10.9745/GHSP-D-20-00362 33795367 PMC8087437

[42] Afulani PA Phillips B Aborigo RA Moyer CA. Person-centred maternity care in low-income and middle-income countries: analysis of data from Kenya, Ghana, and India. Lancet Glob Health. (2019) 7(1):e96–e109. 10.1016/S2214-109X(18)30403-0 30554766 PMC6293963

[43] Malepe TC Havenga Y Mabusela PD. Barriers to family-centred care of hospitalised children at a hospital in Gauteng. Health SA Gesondheid (Online). (2022) 27:1–10. 10.4102/hsag.v27i0.1786 PMC9634677 36337442

[44] Salehi R Asamoah A de Young S Acquah H Agarwal N Aryee SE Scaling up pediatric nurse specialist education in Ghana—a longitudinal, mixed methods evaluation. BMC Nurs. (2021) 20(1):32. 10.1186/s12912-021-00550-1 33593320 PMC7885484

[45] Baker U Hassan F Hanson C Manzi F Marchant T Swartling Peterson S Unpredictability dictates quality of maternal and newborn care provision in rural Tanzania-A qualitative study of health workers’ perspectives. BMC Pregnancy Childbirth. (2017) 17(1):55. 10.1186/s12884-017-1230-y 28166745 PMC5294891

[46] Leslie HH Sun Z Kruk ME. Association between infrastructure and observed quality of care in 4 healthcare services: a cross-sectional study of 4,300 facilities in 8 countries. PLoS Med. (2017) 14(12):e1002464. 10.1371/journal.pmed.1002464 29232377 PMC5726617

[47] Mocumbi S McKee K Munguambe K Chiau R Högberg U Hanson C Ready to deliver maternal and newborn care? Health providers’ perceptions of their work context in rural Mozambique. Glob Health Action. (2018) 11(1):1532631. 10.1080/16549716.2018.1532631 30387378 PMC6225433

[48] Geleto A Chojenta C Musa A Loxton D. Barriers to access and utilization of emergency obstetric care at health facilities in sub-Saharan Africa: a systematic review of literature. Syst Rev. (2018) 7(1):183. 10.1186/s13643-018-0842-2 30424808 PMC6234634

[49] Kweku M Amu H Awolu A Adjuik M Ayanore MA Manu E Community-based health planning and services plus programme in Ghana: a qualitative study with stakeholders in two systems learning districts on improving the implementation of primary health care. PLoS One. (2020) 15(1):e0226808. 10.1371/journal.pone.0226808 31914122 PMC6948830

[50] Elsey H Abboah-Offei M Vidyasagaran AL Anaseba D Wallace L Nwameme A Implementation of the community-based health planning and services (CHPS) in rural and urban Ghana: a history and systematic review of what works, for whom and why. Front Public Health. (2023) 11:1105495. 10.3389/fpubh.2023.1105495 37435526 PMC10332345

[51] Stokes T Shaw EJ Camosso-Stefinovic J Imamura M Kanguru L Hussein J. Barriers and enablers to guideline implementation strategies to improve obstetric care practice in low- and middle-income countries: a systematic review of qualitative evidence. Implementation Science: iS. (2016) 11(1):144. 10.1186/s13012-016-0508-1 27770807 PMC5075167

[52] Oduro-Mensah E Agyepong IA Frimpong E Zweekhorst M Vanotoo LA. Implementation of a referral and expert advice call center for maternal and newborn care in the resource constrained health system context of the greater Accra region of Ghana. BMC Pregnancy Childbirth. (2021) 21(1):56. 10.1186/s12884-020-03534-2 33441115 PMC7807452

[53] Kushitor MK Biney AA Wright K Phillips JF Awoonor-Williams JK Bawah AA. A qualitative appraisal of stakeholders’ perspectives of a community-based primary health care program in rural Ghana. BMC Health Serv Res. (2019) 19(1):N.PAG–N.PAG. 10.1186/s12913-019-4506-2 PMC6751899 31533696

[54] Gondwe MJ Desmond N Aminu M Allen S. Resource availability and barriers to delivering quality care for newborns in hospitals in the southern region of Malawi: a multisite observational study. PLOS Global Public Health. (2022) 2(12):e0001333. 10.1371/journal.pgph.0001333 36962885 PMC10021306

[55] Lasim OU Ansah EW Apaak D. Maternal and child health data quality in health care facilities at the Cape Coast Metropolis, Ghana. BMC Health Serv Res. (2022) 22(1):1102. 10.1186/s12913-022-08449-6 36042447 PMC9425804

[56] Bradley S McCourt C Rayment J Parmar D. Midwives’ perspectives on (dis)respectful intrapartum care during facility-based delivery in Sub-Saharan Africa: a qualitative systematic review and meta-synthesis. Reprod Health. (2019) 16(1):116. 10.1186/s12978-019-0773-y 31345239 PMC6659209

[57] Aikins DA Poku CA Donkor E Naab F. Practice environment determinants of job satisfaction among midwives at healthcare facilities in Accra Metropolis: a multicentre study. PLoS One. (2023) 18(3):e0282251. 10.1371/journal.pone.0282251 36857327 PMC9977032

[58] Haider S Ali RF Ahmed M Humayon AA Sajjad M Ahmad J. Barriers to implementation of emergency obstetric and neonatal care in rural Pakistan. PLoS One. (2019) 14(11):e0224161. 10.1371/journal.pone.0224161 31689316 PMC6830770

[59] Pfeiffer E Owen M Pettitt-Schieber C Van Zeijl R Srofenyoh E Olufolabi A Building health system capacity to improve maternal and newborn care: a pilot leadership program for frontline staff at a tertiary hospital in Ghana. BMC Med Educ. (2019) 19(1):52. 10.1186/s12909-019-1463-8 30744625 PMC6371505

[60] Toivonen M Lehtonen L Ahlqvist-Björkroth S Axelin A. Key factors supporting implementation of a training program for neonatal family- centered care—a qualitative study. BMC Health Serv Res. (2019) 19(1):394. 10.1186/s12913-019-4256-1 31217007 PMC6585011

[61] Ayanore MA Asampong R Alhassan RK Doegah P Acquah E Kugbey N Informal payments and willingness to pay informally for health care among older adults: equity perspectives for geriatric care in Ghana. J Glob Health Sci. (2023) 5(1):1–15. 10.35500/jghs.2023.5.e7

[62] Dalaba MA Welaga P Immurana M Ayanore M Ane J Danchaka LL Cost of childbirth in upper west region of Ghana: a cross-sectional study. BMC Pregnancy Childbirth. (2022) 22(1):613. 10.1186/s12884-022-04947-x 35927635 PMC9351074

[63] Puchalski Ritchie LM Khan S Moore JE Timmings C van Lettow M Vogel JP Low- and middle-income countries face many common barriers to implementation of maternal health evidence products. J Clin Epidemiol. (2016) 76:229–37. 10.1016/j.jclinepi.2016.02.017 26931284

[64] Ministry of Health G. National Health Policy: Ensuring Healthy Lives for all. Accra: Ministry of Health (2020). Available at: https://extranet.who.int/countryplanningcycles/sites/default/files/public_file_rep/GHA_Ghana_National-Health-Policy-NHP_2020.pdf

